# Contact heat evoked potentials and habituation measured interictally in migraineurs

**DOI:** 10.1186/1129-2377-16-1

**Published:** 2015-01-06

**Authors:** Lena Clara Beese, Denise Putzer, Nani Osada, Stefan Evers, Martin Marziniak

**Affiliations:** 1 Department of General Neurology, University of Münster, Albert-Schweitzer-Campus 1, 48149 Münster, Germany; 2 Department of Medical Informatics and Biomathematics, University of Muenster, Albert-Schweitzer-Campus 1, 48149 Münster, Germany; 3 Krankenhaus Lindenbrunn, Lindenbrunn 1, 31863 Coppenbrügge, Germany; 4 Department of Neurology, Isar-Amper-Clinic, Munic-East, Ringstrasse 56A, 85540 Haar, Germany

**Keywords:** Contact heat evoked potentials, Habituation, Migraine, Quantitative sensory testing

## Abstract

**Background:**

A lack of habituation of different evoked potential modalities in migraine patients in-between attacks has been suggested.

**Methods:**

This study investigates cortical response after painful stimuli evaluated by contact heat evoked potentials (CHEPs) and quantitative sensory testing (QST) during the migraine-free interval. We enrolled 22 migraine patients and 22 healthy subjects.

**Results:**

Cortical potentials after contact heat stimulation of the cheeks and the volar forearm at a temperature of 51°C showed significantly reduced A-δ-amplitudes in patients and healthy controls. When the subjects’ attention was drawn to an arithmetic task, a partial lack of habituation of amplitude could be seen in migraine patients. QST did not show any difference between migraineurs and controls.

**Conclusion:**

Our findings can be primarily deemed to demonstrate that patients and healthy controls show significantly lower amplitudes while performing the calculation task. Without performing the calculation task we could not show the expected lack of habituation in migraineurs. Yet, while performing the calculation task our results partly suggest that hypothesis.

## Background

In migraine, a characteristic neurophysiological finding during the interictal phase is a lack of habituation of evoked potentials of different modalities to repeated stimuli in comparison to healthy subjects [1]. According to Rankin et al. habituation is defined as "a behavioral response decrement that results from repeated stimulation" [2]. A deficient habituation of evoked potentials during the pain-free interval of migraine patients has been shown for nonpainful visual [3], auditory [4], somatosensory [5] and nociceptive stimuli [6] as well as for brainstem reflexes [7]. It is proposed that thalomocortical dysrhythmia may be responsible for altered synchronicity in migraine [8, 9]. In contrast, the habituation deficit could not always be affirmed: Sand et al. and Omland et al. failed to establish any habituation difference for nonpainful visual evoked potentials between migraine patients and healthy subjects [10, 11]. Moreover, it was shown that clinical fluctuations are correlated to specific patterns of somatosensory evoked potential habituation with stable N20 responses in improving patients [12].

Interictal lack of habituation to experimental pain has been demonstrated with noxious laser stimuli [13]. Contact heat evoked potentials (CHEPs) using a contact thermode that rapidly increases skin temperature have recently been introduced to study nociceptive pathways [14]. The main advantage of CHEPs compared to LEPs is that reliable scalp potentials can be obtained without cutaneous lesions. Also, they do not require any precautions such as safety glasses [15]. So far, however, few studies have been carried out applying CHEPs to investigate habituation in migraine explicitly focusing on EEG-tomography to identify the cortical mechanisms underlying the processes of habituation to experimental pain. In two studies by Lev et al. reduced inhibitory functioning of the prefrontal cortex is suggested as a possible cause for disinhibition of the pain-related sensory cortices in migraine [16, 17].

Quantitative sensory testing (QST) according to the protocol of the German Research Network on Neuropathic Pain (Deutscher Forschungsverbund Neuropathischer Schmerz, DFNS) consists of a standardized battery of sensory examinations [18]. It provides useful information about pain thresholds as it allows to quantify thermal allodynia and other hyperalgesic conditions [19]. Burstein discovered an increased cutaneous allodynia in acute migraine attacks in 79% of the patients suggesting that the pathophysiology of migraine involves a sensitization of central neurons [20]. There are contradictory results about interictal pain thresholds in migraine patients compared to healthy subjects: In some studies, no difference in thermal, pressure and electrical pain or detection thresholds and no allodynia could be found [21].

Therefore, our aim was to investigate interictal changes in the sensory state of neuronal pathways in migraine patients. The objective of the present study was to explore the behavior of the subjective pain sensation during repetitive painful stimulation by CHEPs in relation to the amplitude modifications of the cortical evoked potentials. This behavior was studied both during attention towards the stimulus and a distraction task. The main research question was: Do migraine patients show impaired habituation of CHEPs? Moreover, our aim was to investigate whether in migraine patients attention cannot easily be drawn away from induced pain by distraction. We combined the study with QST to evaluate if physiological thresholds and the absence of neuropathic pain can be used as a vantage point for the usability of the CHEPs data.

## Methods

We included 22 female patients (23.64 ± 4.30 years) suffering from migraine with or without aura with at least one migraine attack per month (Table 1). Before starting the study a sample size estimation was performed, based on an estimated reduction of amplitude of 30% and a power of 80%, which suggested a sample size of 22. The calculation was based on the CHEPs data of Suttrup et al. [22]. The diagnosis was made according to the criteria of the International Classification of Headache Disorders 2^nd^ edition [23]. Patients did not complain about other neurological disorders and were not taking any pain or sensory modulating drugs. The control group included 22 aged-matched healthy female controls (24.09 ± 3.78 years, Table 1). As it is known that the menstrual cycle may influence the subjective pain thresholds, we kept record of the current menstrual cycle day [24]. Patients were at a mean cycle day of 15.52 ± 7.94 and healthy subjects 14.55 ± 7.87 representing all episodes of the cycle. Patients and probands enrolled in the study were mainly students from all fields. We did not recruit patients from a headache clinic because we set value on examining patients who did not have a chronic migraine. Subjects with general medical, neurological or psychiatric diseases or patients who had taken analgesics during the last 48 hours were excluded from the study. We only included patients who were not taking centrally acting drugs or prophylactic treatment for migraine. Migraine patients were tested at least 48 hours after the last attack and patients were excluded from the analysis if they experienced a migraine attack within 48 hours of the test. The occurrence of migraine attacks was monitored using a headache calendar filled out by the patients beginning one week before the recording and by contacting the patients three days after the recording. Two migraine patients were excluded because a migraine attack occurred less than 48 hours after the recording session.

**Table 1:** **Clinical data of migraine patients and healthy controls**

	Migraine patients	Healthy controls
Age (years)	23.64 ± 4.30	24.09 ± 3.78
STAI-G X1	31.82 ± 5.01	30.55 ± 5.70
STAI-G X2	33.41 ± 7.61	31.73 ± 4.41
Handedness	20 right, 2 left	19 right, 3 left
Day of menstrual cycle	15.52 ± 7.94	14.55 ± 7.87
Disease duration (years)	8.95 ± 4.61	
Headache side during migraine	14 right, 8 left	
Attacks per month	2.17 ± 2.86	
Aura	12 with, 10 without	
Family history	13 positive, 9 negative	
Intensity of headache during untreated attack (0–10)	7.00 ± 1.11	
Accessory symptoms during migraine attack:		
Nausea	21 (95%)	
Vomiting	14 (64%)	
Sensitivity to light	19 (86%)	
Sensitivity to noise	21 (95%)	
Sensitivity to odors	8 (36%)	
Intensification through activity	18 (82%)	
Restriction of daily activities	20 (91%)	
One-sided headache	21 (95%)	
Pulsatile headache	19 (86%)	

Mean group values ± standard deviation for 22 migraine patients and 22 healthy controls.

To evaluate the emotional condition of the patients, we used the State-Trait-Anxiety-Inventory. This test describes the level of state anxiety (STAI-G X1) and the level of trait anxiety (STAI-G X2) [25]. The study was performed in accordance with the Helsinki Declaration. Informed consent was obtained from each subject before the commencement of our study.

### Quantitative sensory testing

We used a modified standardized QST-battery developed by the DFNS in order to detect loss or gain of sensory function [18]. The stimulation area for the QST was the volar forearm and the cheek bilaterally in both patients and healthy controls.

A peltier-based contact probe of 30 mm for the volar forearm and 16 mm for the cheek was used to investigate C and A-δ-fiber function, employing the ATS-system (Medoc Ltd., Ramat Yishai, Israel). We determined cold and warm detection thresholds (CDT, WDT), as well as cold and heat pain thresholds (CPT, HPT). Ramped stimuli were used to obtain thresholds: 1°C/s for cold and warm thresholds, 1.5°C/s for the pain thresholds, starting from a reference temperature of 32°C. The subjects had to press a button as soon as they perceived a change in temperature (CDT, WDT) or a painful sensation (CPT, HPT). Temperature limits were 0°C and 50°C respectively. In order to evaluate the mean threshold temperature each threshold was assessed three times.

A standardized set of modified "von Frey filaments" (0.25–512 mN) was used to determine the mechanical detection threshold (MDT) to quantify the A-β-fiber function. A brush (200–400 mN) which was applied three times was employed to examine pain in response to light touch (dynamic mechanical allodynia). The mechanical pain threshold (MPT) was tested with a set of seven standardized pinprick stimulators (8–512 mN). Both patients and healthy controls were asked to define the pain they perceived as either "blunt" or "sharp". Five infra- and five suprathreshold values were obtained and the geometric mean was determined.

The perceptual wind-up ratio (WUR) was tested by a single pinprick stimulus (256 mN) followed by a train of ten repetitive stimuli of the same intensity and area. Subjects were requested to compare the intensity of the initial stimulus with the ten stimuli using the NRS. The test was repeated five times and the WUR was calculated by dividing the mean pain rating of five series by the mean pain rating of five single stimuli.

The test battery was performed in the same order using the same equipment and standardized instructions for all patients and healthy subjects [18].

### Contact heat evoked potentials

A CHEP stimulator (Medoc Ltd., Ramat Yishai, Israel) with an accelerated velocity of 70°C/s and a cooling rate of 40°C/s was used to deliver cutaneous heat stimuli. Twenty constant-intensity stimuli of 51°C (baseline temperature 32°C) were applied to the same body region with an inter-stimuli interval of 15–18 seconds in each stimulation block. The thermode was based on a peltier element (target area of 573 mm^2^). The contact heat stimulation was performed in a randomized order on the glabrous skin of the volar forearm, on the right and on the left cheek. In migraine patients, we used the arm of the body side where the migraine attacks usually occurred; in healthy subjects we used the side of the arm matching to the corresponding migraine patient. Every examination was followed by a second trial, resulting in a total duration of 50 minutes. After a 10-minute break, the same procedure involving 20 stimuli applied twice in three areas was repeated while the subject had to perform mental arithmetic tasks consisting of subtractions of a 3-digit number with crossing the tens barrier. The arithmetic task was given about 5 seconds before each stimulus. The result had to be announced by the subject after the stimulus [26].

Keypoint system (Medtronic, Skovlunde, Denmark) was used to record CHEPs. The potentials were recorded from P3/P4 for the contralateral cheek and from CZ’ for the arm with the reference electrode on the forehead using silver/silver chloride cup electrodes with a 9 mm cup diameter filled with a conductive adhesive gel. Four single waves, consisting of five averaged stimuli each, were obtained in each train of the stimulation. Latencies were measured from the first definitive negative peak (N2) and the amplitude from peak to peak (N2 to P2) within each waveform. Since CHEPs is very suitable for A-δ potentials recording, we concentrated on the A-δ-mediated late potential (N2) instead of the ultra-late C-fiber mediated potential [27].

**Figure 1:**
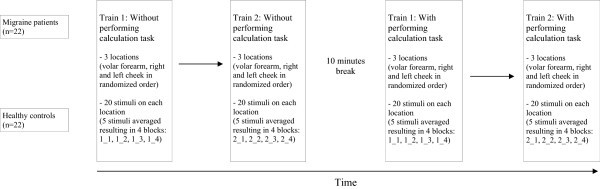
**Study design of the contact heat evoked potentials stimulation.**

Subjects had to lie on their back with their eyes closed and were requested to relax completely during the examination. Patients and healthy controls were told to rate their acute pain on a 0–10 level numerical rating scale (NRS) after each stimulus, with 0 equaling "no pain" and 10 "maximum imaginable pain". This protocol was applied for patients and healthy controls alike. Figure 1 shows a time flow to visualize the study design.

A similar setting and the same methods of QST and CHEPs have been used in a study examining idiopathic dystonia [22].

### Data evaluation

Clinical data, CHEPs and QST parameters were evaluated using SPSS Software Release 18.0. The continuous variables CHEPs parameters of the first and second train without and with performing the calculation task (amplitudes of block 1, 2, 3 and 4 and N2 wave latencies of the volar forearm, right and left cheek), QST parameters (CDT, WDT, CPT, HPT, MDT, MPT and WUR) and log data of QST parameters were expressed as mean ± standard deviation (SD), and the interquartile range [25^th^ percentile; 75^th^ percentile] only for CHEPs parameters, whereas categorical variables were expressed as frequency and percentage (see Tables 1, 2, 3 and 4).

**Table 2:** **Mean, standard deviation (SD) and interquartile range (IQR) [25** ^**th**^ **percentile; 75** ^**th**^ **percentile] for the amplitude of contact heat evoked potentials of the volar forearm (2a) and the pooled cheek (right and left cheek, 2b) of migraine patients and healthy controls for trains without and with performing the calculation task for blocks 1 to 4 of train 1 and train 2**

**a**						
**Volar forearm without performing the calculation task**						
	**Migraine patients**			**Healthy controls**		
**Amplitude (μV)**	**Mean**	**SD**	**IQR**	**Mean**	**SD**	**IQR**
Ampl_1_1	56.33	22.38	[48.80; 77.50]	66.09	24.93	[51.38; 79.28]
Ampl_1_2	48.57	25.73	[31.00; 76.70]	56.21	24.58	[40.93; 75.82]
Ampl_1_3	43.78	22.73	[29.20; 58.10]	51.11	27.48	[41.10; 55.10]
Ampl_1_4	43.32	22.27	[29.60; 61.10]	47.39	22.92	[35.08; 66.35]
Ampl_2_1	47.55	20.33	[33.30; 98.50]	57.36	26.81	[47.20; 80.05]
Ampl_2_2	42.30	18.59	[28.36; 59.80]	48.51	24.06	[40.58; 59.03]
Ampl_2_3	43.15	20.76	[24.75; 63.30]	47.32	23.96	[34.20; 65.10]
Ampl_2_4	36.50	18.02	[25.20; 48.60]	49.05	23.59	[28.88; 58.75]
p value of train 1 (power)	p = 0.006 (0.74)	chi-square = 9.579; df = 2		p < 0.001 (0.94)	chi-square = 20.667; df = 2	
p value of train 2 (power)	p = 0.001 (0.73)	chi-square = 13.875; df = 2		p = 0.001 (0.32)	chi-square = 14.632; df = 2	
**Volar forearm with performing the calculation task**						
	**Migraine patients**			**Healthy controls**		
**Amplitude (μV)**	**Mean**	**SD**	**IQR**	**Mean**	**SD**	**IQR**
Ampl_1_1	35.80	13.60	[32.20; 57.45]	39.96	29.61	[21.04; 60.38]
Ampl_1_2	34.75	15.33	[32.42; 59.58]	39.69	29.23	[21.37; 55.70]
Ampl_1_3	29.56	13.21	[21.52; 47.58]	34.43	24.98	[20.99; 55.75]
Ampl_1_4	30.30	11.85	[26.63; 48.80]	30.95	25.74	[16.93; 50.00]
Ampl_2_1	36.80	13.41	[32.99; 47.63]	44.87	35.63	[23.51; 52.35]
Ampl_2_2	32.15	12.41	[23.38; 44.78]	39.04	25.35	[23.27; 56.53]
Ampl_2_3	32.40	11.27	[26.82; 44.25]	31.29	27.05	[20.11; 45.88]
Ampl_2_4	29.78	8.98	[22.26; 38.48]	42.24	26.86	[18.72; 66.38]
p value of train 1 (power)	p = 0.176 (0.49)	chi-square = 4.000; df = 2		p = 0.018 (0.31)	chi-square = 9.385; df = 2	
p value of train 2 (power)	p = 0.199 (0.80)	chi-square = 4.500; df = 2		p = 0.662 (0.06)	chi-square = 1.500 ; df = 2	
**b**						
**Pooled cheek without performing the calculation task**						
	**Migraine patients**			**Healthy controls**		
**Amplitude (μV)**	**Mean**	**SD**	**IQR**	**Mean**	**SD**	**IQR**
Ampl_1_1	53.07	20.41	[38.44; 68.10]	55.91	24.72	[41.77; 69.05]
Ampl_1_2	44.85	17.99	[31.56; 54.63]	43.74	16.83	[32.04; 50.83]
Ampl_1_3	41.76	18.05	[29.98; 49.60]	44.82	18.82	[34.23; 54.35]
Ampl_1_4	39.15	22.90	[27.27; 49.00]	40.24	14.69	[29.85; 51.39]
Ampl_2_1	40.20	15.53	[27.89; 55.10]	44.00	18.43	[30.20; 59.70]
Ampl_2_2	35.38	12.89	[27.64; 44.45]	38.84	13.15	[27.30; 47.70]
Ampl_2_3	36.61	16.55	[24.59; 46.40]	36.80	11.86	[30.60; 40.70]
Ampl_2_4	34.10	14.50	[21.94; 44.77]	36.47	14.57	[25.51; 48.70]
p value of train 1 (power)	p < 0.001 (0.99)	chi-square = 24.057; df = 2		p = 0.001 (0.999)	chi-square = 15.895; df = 2	
p value of train 2 (power)	p = 0.007 (0.75)	chi-square = 9.235; df = 2		p = 0.038 (0.84)	chi-square = 6.359; df = 2	
**Pooled cheek with performing the calculation task**						
	**Migraine patients**			**Healthy controls**		
**Amplitude (μV)**	**Mean**	**SD**	**IQR**	**Mean**	**SD**	**IQR**
Ampl_1_1	31.72	11.30	[25.40; 44.25]	36.16	21.46	[20.74; 53.10]
Ampl_1_2	28.53	9.90	[25.49; 38.85]	31.83	14.08	[21.04; 42.55]
Ampl_1_3	25.83	9.29	[20.68; 37.10]	30.08	12.77	[19.44; 44.19]
Ampl_1_4	26.71	6.26	[22.39; 32.25]	26.76	8.37	[19.95; 34.27]
Ampl_2_1	34.73	12.22	[24.20; 46.40]	32.07	18.22	[18.80; 49.00]
Ampl_2_2	31.42	9.38	[27.28; 38.30]	27.17	11.60	[19.51; 69.91]
Ampl_2_3	27.87	8.27	[20.30; 34.00]	26.75	11.90	[18.88; 37.80]
Ampl_2_4	25.45	10.05	[16.33; 32.10]	25.66	10.29	[19.92; 34.87]
p value of train 1 (power)	p = 0.401 (0.96)	chi-square = 3.000; df = 2		p = 0.010 (1.00)	chi-square = 8.957; df = 2	
p value of train 2 (power)	p < 0.001 (0.999)	chi-square = 16.300; df = 2		p = 0.287 (0.70)	chi-square = 3.111; df = 2	

The p value and power for each train is presented for block 1 vs. block 4.

Statistical comparison with Friedman test for multiple pairwise comparison (blocks 1, 2 and 4), p < 0.05 is considered to be significant.

**Table 3:** **Mean and standard deviation (SD) for the N2 wave latencies of contact heat evoked potentials of the volar forearm and pooled cheek of migraine patients and healthy controls for trains without and with performing the calculation task**

N2 wave latencies (ms)								
	Migraine patients		Healthy controls		p values (power)		Mann–Whitney-***U***	
Without performing the calculation task	Volar forearm (mean ± SD)	Pooled cheek (mean ± SD)	Volar forearm (mean ± SD)	Pooled cheek (mean ± SD)	Volar forearm	Pooled cheek	Volar forearm	Pooled cheek
Train 1	419.90 ± 34.79	333.66 ± 36.15	394.57 ± 23.19	318.95 ± 36.97	0.013 (0.81)	0.122 (0.46)	121.5	669.5
Train 2	416.46 ± 38.53	334.60 ± 32.81	393.32 ± 22.69	313.02 ± 41.76	0.023 (0.69)	0.016 (0.77)	130.0	627.5
**With performing the calculation task**								
Train 1	400.69 ± 34.17	329.56 ± 42.40	365.76 ± 47.11	310.13 ± 39.18	0.046 (0.80)	0.087 (0.60)	98.5	465.0
Train 2	390.92 ± 37.03	325.16 ± 32.18	353.54 ± 48.95	304.61 ± 43.33	0.015 (0.29)	0.045 (0.71)	74.0	321.0

Statistical comparison with Mann–Whitney-*U* test (migraine patients vs. healthy controls), p < 0.05 is considered to be significant.

**Table 4:** **Mean and standard deviation (SD) for QST parameters of the volar forearm, the right and left cheek of patients with migraine and of healthy controls**

Quantitative sensory testing												
	Migraine patients			Healthy controls			p values			Mann–Whitney-***U***		
QST parameter	Arm	Right cheek	Left cheek	Arm	Right cheek	Left cheek	Arm	Right cheek	Left cheek	Arm	Right cheek	Left cheek
	Raw data (mean ± SD)	Raw data (mean ± SD)	Raw data (mean ± SD)	Raw data (mean ± SD)	Raw data (mean ± SD)	Raw data (mean ± SD)						
CDT (°C)	-1.53 ± 0.95	-1.88 ± 1.08	-1.91 ± 0.75	-1.71 ± 1.22	-1.31 ± 0.53	-2.09 ± 0.90	0.743	0.085	0.443	207.5	152.0	190.0
WDT (°C)	1.86 ± 0.78	2.20 ± 1.27	2.62 ± 0.99	1.92 ± 0.52	1.92 ± 0.66	2.42 ± 1.26	0.262	0.990	0.320	176.0	220.0	190.5
CPT (°C)	15.02 ± 6.46	13.38 ± 8.44	12.62 ± 8.97	14.23 ± 6.72	10.25 ± 8.37	11.02 ± 8.13	0.697	0.314	0.450	205.0	180.5	190.5
HPT (°C)	43.08 ± 2.55	44.02 ± 3.15	44.50 ± 3.31	43.10 ± 3.31	44.16 ± 2.86	43.82 ± 2.69	0.782	0.811	0.141	209.5	211.0	162.0
MDT (mN)	3.75 ± 6.18	0.29 ± 0.17	0.32 ± 0.3	2.43 ± 2.49	0.26 ± 0.13	0.26 ± 0.10	0.290	0.919	0.361	178.5	216.5	184.5
MPT (mN)	11.16 ± 10.97	24.34 ± 29.59	14.01 ± 12.45	8.77 ± 5.23	18.50 ± 18.5	10.36 ± 10.8	0.353	0.910	0.073	184.0	216.0	149.5
WUR (ratio)	3.83 ± 2.75	3.88 ± 3.99	4.96 ± 10.14	4.62 ± 6.97	3.62 ± 3.28	4.28 ± 4.36	0.580	0.473	0.615	198.5	192.0	200.5
	**Migraine patients**			**Healthy controls**			**p values**			**t; df**		
**QST parameter**	**Arm**	**Right cheek**	**Left cheek**	**Arm**	**Right cheek**	**Left cheek**	**Arm**	**Right cheek**	**Left cheek**	**Arm**	**Right cheek**	**Left cheek**
	Log data (mean ± SD)	Log data (mean ± SD)	Log data (mean ± SD)	Log data (mean ± SD)	Log data (mean ± SD)	Log data (mean ± SD)						
CDT (°C)	0.13 ± 0.22	0.21 ± 0.23	0.25 ± 0.18	0.14 ± 0.29	0.06 ± 0.28	0.28 ± 0.21	0.897	0.062	0.650	t = 2.628; df = 40	t = 0.334; df = 40	t = 0.433; df = 40
WDT (°C)	0.24 ± 0.15	0.29 ± 0.22	0.39 ± 0.16	0.26 ± 0.14	0.26 ± 0.16	0.35 ± 0.17	0.629	0.632	0.427	t = 0.052; df = 40	t = 0.966; df = 40	t = 0.029; df = 40
MDT (mN)	0.35 ± 0.4	-0.59 ± 0.19	-0.58 ± 0.24	0.21 ± 0.41	-0.61 ± 0.16	-0.61 ± 0.13	0.265	0.709	0.677	t = 0.163; df = 40	t = 0.646; df = 40	t = 4.196; df = 30.162
MPT (mN)	0.96 ± 0.24	1.21 ± 0.37	1.04 ± 0.27	0.90 ± 0.19	1.15 ± 0.29	0.93 ± 0.23	0.378	0.609	0.138	t = 0.264; df = 40	t = 3.002; df = 40	t = 0.832; df = 40
WUR (ratio)	0.50 ± 0.27	0.47 ± 0.29	0.42 ± 0.44	0.47 ± 0.35	0.50 ± 0.31	0.50 ± 0.31	0.811	0.683	0.503	t = 0.433; df = 40	t = 0.057; df = 40	t = 0.094; df = 40

QST data are shown as log_10_ units (lg) except for the cold and heat pain thresholds (raw data) and as raw data. Statistical comparison of raw data with Mann–Whitney-*U* test (migraine patients vs. healthy controls) and statistical comparison of log data with independent t-test, p < 0.05 is considered to be significant. None of the subjects showed dynamic mechanical allodynia.

Before statistical testing, the Kolmogorov-Smirnov test was used to analyze each continuous variable for its normal distribution. If the samples were not normally distributed, the non-parametric test was used. The Mann–Whitney-*U* test was used to compare the independent variables between two study groups (amplitude differences, absolute reduction of amplitude, success rate of calculating, N2 wave latencies, QST parameters); the Wilcoxon test was used to compare the dependent variables between study groups (two different localizations of CHEPs and QST parameters). The Friedman signed rank test was used to compare the time-dependent variables of each groups (amplitude of block 1 to 4 for migraine patients and healthy controls). Pairwise multiple comparisons with the Friedman test were performed using the Dunn-procedure [28, 29]. We used the independent samples *t*-test to compare the parametric log data of the QST parameters between study groups. The Pearson correlation coefficient was used to evaluate the relationship between the HPT and the N2 wave latencies and the relationship between amplitudes and numerical rating scale. Differences were considered statistically significant at p < 0.05. PS program and Java-Applets of JUMBO were used to perform power calculations [30, 31].

As QST data were log-normally distributed, they were expressed as log_10_, except for the CPT and HPT, which are normally distributed as raw data. We used the *z*-transformation to present all QST-parameters of each healthy control as standard normal distribution in order to compare a single patient’s data profile with the control group without considering the different units of measurement across QST-parameters: *z*-score = (X_single patient_ - Mean_controls_)/SD_controls_. All QST-parameters were represented as a normal standard distribution with consideration of gender and age, based on this calculation. *Z*-values below -2 and above 2 are considered pathological [18]. Moreover, we used the Mann–Whitney-*U* test to compare patients with controls.

## Results

The clinical characteristics of the patients and subjects are shown in Table 1. There were no significant differences in the level of state anxiety (p = 0.334) or in the level of trait anxiety (p = 0.991) between migraine patients and healthy controls.

### QST

**Figure 2:**
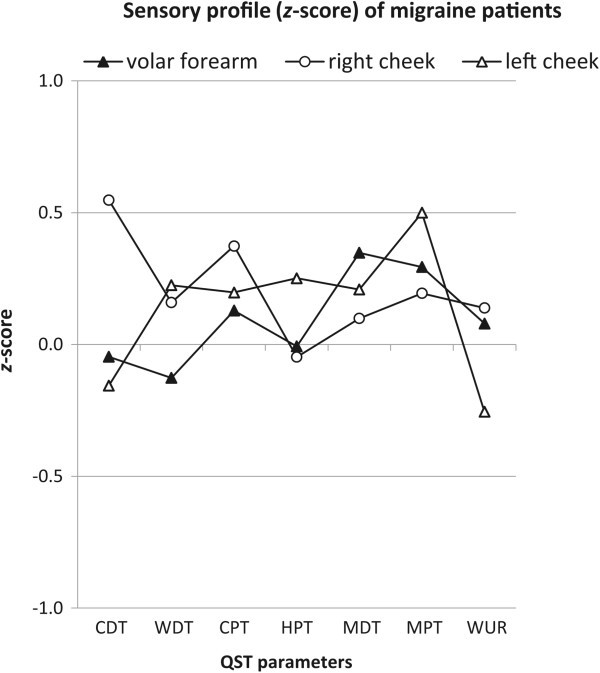
**QST sensory profile of the volar forearm, right and left cheek of patients with migraine.** The *z*-values were calculated based on the age-matched healthy control group (represented by the zero line). Data are shown as group means. CDT, cold detection threshold; WDT, warm detection threshold; CPT, cold pain threshold; HPT, heat pain threshold; MDT, mechanical detection threshold; MPT, mechanical pain threshold; WUR, wind-up ratio.

The mean values of the QST parameters measured at the volar forearm and both cheeks are shown in Table 4 as well as in the sensory profile represented by the *z*-score (Figure 2). All parameters were within the normal range. None of the patients or healthy subjects showed any sign of dynamic mechanical allodynia. There were no significant differences for any parameter (neither for the raw nor the log data) between patients and healthy controls (Mann–Whitney-*U* test, p > 0.05).

### CHEPs

**Figure 3:**
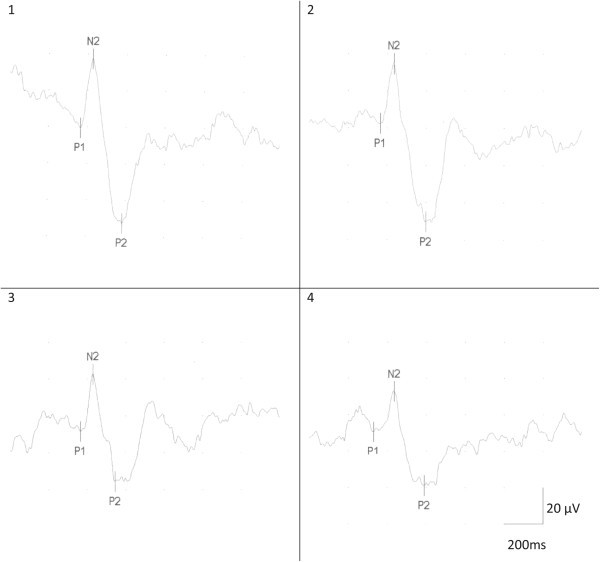
**Contact heat evoked potentials of a patient’s right volar forearm of block 1 (stimulation 1 to 5, N2 P2 amplitude 89.80 μV, N2 wave latency 425.00 ms), block 2 (stimulation 6 to 10, N2 P2 amplitude 84.90 μV, N2 wave latency 435.00 ms), block 3 (stimulation 11 to 15, N2 P2 amplitude 58.10 μV, N2 wave latency 432.00 ms) and block 4 (stimulation 16 to 20, N2 P2 amplitude 51.90 μV, N2 wave latency 435.00 ms) without performing the calculation task, train 1.**

A clear heat pain evoked potential in patients and healthy controls was produced by stimulation of the volar forearm and the cheeks. Figure 3 shows a representative heat pain evoked potential of a patient’s volar forearm. No significant side difference was found between the left and right cheek when average amplitudes were calculated (Wilcoxon test, p > 0.05). Moreover, there was no difference in amplitude or habituation in migraine patients between the cheek of the side where the migraine attack occurs and the other side. For this reason, the amplitude of the right and left cheeks were computed as summary measures and analyzed statistically as "pooled cheek". There were no significant average amplitude differences between migraine patients with or without aura (Mann–Whitney-*U* test, p > 0.05).

**Figure 4:**
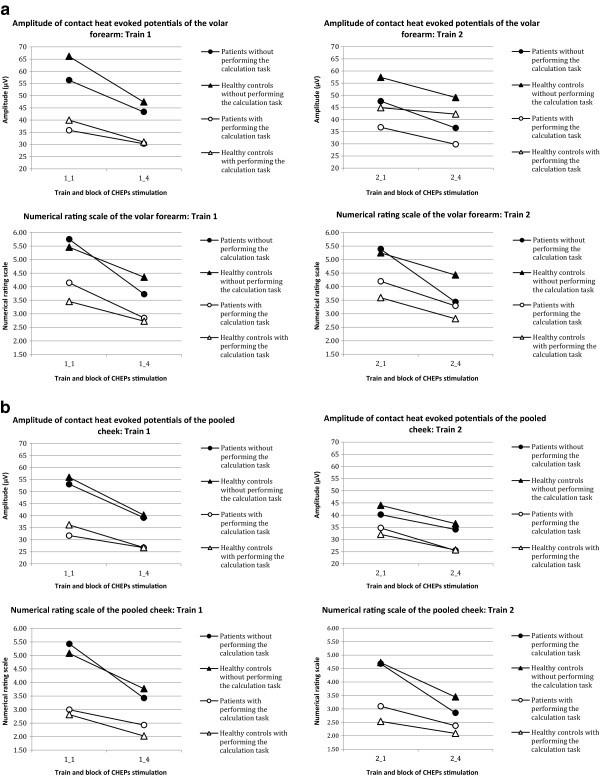
**Development of the amplitude from block 1 to block 4 of the volar forearm and the pooled cheek in relation to the numerical rating scale. a**: Amplitude of the volar forearm of patients with migraine and healthy controls with and without performing the calculation task during contact heat evoked potential stimulation in relation to the numerical rating scale of the volar forearm. Data are shown as group means. Break of about 10 minutes between 1_4 and 2_1. **b**: Amplitude of the pooled cheek of patients with migraine and healthy controls with and without performing the calculation task during contact heat evoked potential stimulation in relation to the numerical rating scale of the pooled cheek. Data are shown as group means. Break of about 10 minutes between 1_4 and 2_1.

Both patients and controls showed a significant decrease of amplitudes from the first to the fourth block in train one and train two for both pooled cheeks and arm without performing the calculation task (Table 2a and b). During distraction by the arithmetic task, healthy controls exhibited a significant decrease of amplitude in the first train of the arm and the pooled cheek, but there was no significant reduction in the second train of each location. Patients neither showed significantly reduced amplitudes in both trains of the arm nor in the first train of the pooled cheek. There were no significant differences in the absolute reduction (amplitude block 4 minus amplitude block 1) between patients and subjects, as well as between the first and the second train within the groups. Figure 4 shows the development of the amplitude from block 1 (stimuli 1 to 5) to block 4 (stimuli 16 to 20) of train 1 and train 2 for patients and healthy probands with and without performing the calculation task in relation to the numerical rating scale.

During the arithmetic task, amplitudes were significantly lower in patients and subjects compared to the trains without performing the calculation task (Wilcoxon test, p < 0.05).

There were no differences in the average success rate of calculating: On average, both patients and subjects solved 70% of the arithmetic tasks correctly.

The N2 wave latencies of the migraine patients were significantly longer than the N2 wave latencies of the healthy controls for the arm both without performing the calculation task and with performing the calculation task (Table 3). For the pooled cheek, the N2 wave latencies of the migraine patients were significantly longer without performing the calculation task and with performing the calculation task only for the second train. There were no significance differences for the first train without performing the calculation task or with performing the calculation task, with the tendency remaining as mentioned earlier. There was no correlation between HPT and N2 wave latency. Moreover, there was no correlation between amplitude and numerical rating scale.

Patients and subjects reported a significant decrease of pain intensity for all locations and all trains, with and without performing the calculation task from block 1 to block 4 (p < 0.05) (Figure 4).

## Discussion

As previous studies have already shown, interictal tests of migraine patients do not reveal any pathological pain thresholds or cutaneous allodynia [21, 32, 33]. Allodynia has been demonstrated to be a transient phenomenon [34]. Our results confirm these observations and extend them to detection thresholds. In contrast, Schwedt et al. suggest that migraine patients measured interictally are more sensitive to thermal stimulation than healthy controls [35]. In their study heat and cold pain thresholds measured for controls are remarkably different from current physiological values [18] and therefore create the impression that the migraine patients’ data are pathological. For our study, we could use the physiological thresholds and the absence of neuropathic pain as a vantage point for the usability of the CHEPs data.

Lack of habituation of evoked potentials is regarded as an interictal marker in migraine patients and as an expression of cortical information processing abnormalities [36]. In contrast to most previous studies we could not find any significant lack of diminution of amplitude in migraine patients during the two trains without performing the calculation task for both volar forearm and pooled cheek. Therefore we were unable to demonstrate any abnormal habituation process. During the arithmetic distraction task, however, a lack of habituation could be found for migraine patients in the first train for both locations. In the second train, healthy subjects did not habituate anymore and migraine patients surprisingly demonstrated habituation of amplitude when the pooled cheek was tested.

Our study did not confirm the recently stated hypothesis that in migraine patients attention cannot easily be drawn away from induced pain because patients and healthy controls showed significantly lower amplitudes while performing the calculation task [26]. However, the expected lack of habituation could only be shown during distraction. Further studies will have to be performed concentrating explicitly on the effects of distraction on contact heat evoked potentials to investigate the pathophysiological background.

Lev et al. described a physiological habituation in controls and a potentiation of cortical responses in migraine patients to noxious heat evoked stimuli [16]. In contrast to our study, however, they calculated the inter-train activity by subtracting the first train from the second train (each consisting of 30 averaged stimuli) instead of calculating the amplitude change between the first and the last blocks of averaging [37]. Our results show that averaging five stimuli can already generate a reproducible potential that demonstrates the habituation earlier.

The longer N2 wave latencies in migraine patients cannot be clarified at this point and will have to be subject of further research.

There are a few possible explanations for the unexpected habituation in migraine patients in our study. First, our subjects were young (mean age of healthy subjects 24.09 years, mean age of migraine patients 23.64 years) with a relatively short disease duration (mean disease duration 8.95 years). There was no correlation between the number of attacks and the reduction of amplitude. Moreover, as extra-cephalic hypersensitivity occurs predominantly in severe headache forms [21], it might be that neuronal dysexcitability was too weak in our migraine patients. In addition, we could not consider the effect of clinical fluctuations since our study did not include a clinical follow-up of more than three days. Furthermore, the total duration of the experimental setting is rather long compared to other studies investigating habituation [38]. Therefore the initial CHEPs amplitude in the later trains is not equal to the one at the outset of the study, which might conceal possible habituation effects.

Finally, certain limitations of the present study should be acknowledged. First, since subjects showed differences in their ability to solve arithmetic tasks, we cannot be certain of an absolute distraction. However, there was no correlation between the number of correctly solved arithmetic tasks and the reduction of amplitude. Moreover, we think that 22 subjects in each group is a sufficient number and would not expect results to be any different for any increased number of examined probands. In our final analysis we did not distinguish between patients with or without aura (12 patients with aura, 10 patients without aura), but a subgroup analysis showed that their data did not differ from each other. In addition, the break between train 1 and train 2, which lasted about 10 minutes, might have been too short. The averaged amplitude of the first block of train 2 does not reach the initial level of train 1 and therefore does not leave adequate potential for habituation in the second train. In our study, the thermode was moved slightly after each stimulus to prevent sensitization of the skin. It is possible, though, that this movement impeded the expected lack of habituation. We enrolled only female patients and probands in the study to create a study group as homogenous as possible and to avoid effects influenced by the gender. Moreover, it has been shown that gender of both the experimenter and the subjects has a major impact on the subjective pain thresholds [39]. Nevertheless, using only female subjects might be a limitation of our study. Furthermore, the patients were students and not necessarily enrolled in a headache clinic. Therefore, they possibly did not feeling the same level of discomfort as patients who actively go to a consultation.

## Conclusion

Considering all these aspects, we can mainly make a statement about the attentive paradigm, demonstrating that both patients and subjects show significantly lower amplitudes while performing the calculation task. Without performing the calculation task we could not show the expected lack of habituation in migraine patients. Yet, while performing the calculation task our results partly confirm the hypothesis. Concerning the habituation of pain evoked potentials in migraine patients, further studies will have to be conducted to analyze whether the commonly found lack of habituation in migraine patients might be dependent on the modality of evocation.

## References

[1] Brighina F, Palermo A, Fierro B (2009). Cortical inhibition and habituation to evoked potentials: relevance for pathophysiology of migraine. J Headache Pain.

[2] Rankin CH, Abrams T, Barry RJ, Bhatnagar S, Clayton DF, Colombo J (2009). Habituation revisited: An updated and revised description of the behavioral characteristics of habituation. Neurobiol Learn Mem.

[3] Afra J, Proietti Cecchini A, Sandor PS, Schoenen J (2000). Comparison of visual and auditory evoked cortical potentials in migraine patients between attacks. Clin Neurophysiol.

[4] Sand T, Zhitniy N, White LR, Stovner LJ (2008). Brainstem auditory-evoked potential habituation and intensity-dependence related to serotonin metabolism in migraine: A longitudinal study. Clin Neurophysiol.

[5] Ozkul Y, Uckardes A (2002). Median nerve somatosensory evoked potentials in migraine. Eur J Neurol.

[6] de Tommaso M, Libro G, Guido M, Losito L, Lamberti P, Livrea P (2005). Habituation of single CO2 laser-evoked responses during interictal phase of migraine. J Headache Pain.

[7] Katsarava Z, Giffin N, Diener HC, Kaube H (2003). Abnormal habituation of ‘nociceptive’ blink reflex in migraine–evidence for increased excitability of trigeminal nociception. Cephalalgia.

[8] de Tommaso M, Ambrosini A, Brighina F, Coppola G, Perrotta A, Pierelli F (2014). Altered processing of sensory stimuli in patients with migraine. Nat Rev Neurol.

[9] Coppola G, Di Lorenzo C, Schoenen J, Pierelli F (2013). Habituation and sensitization in primary headaches. J Headache Pain.

[10] Sand T, Zhitniy N, White LR, Stovner LJ (2008). Visual evoked potential latency, amplitude and habituation in migraine: a longitudinal study. Clin Neurophysiol.

[11] Omland PM, Nilsen KB, Uglem M, Gravdahl G, Linde M, Hagen K (2013). Visual evoked potentials in interictal migraine: no confirmation of abnormal habituation. Headache.

[12] Restuccia D, Vollono C, Virdis D, del Piero I, Martucci L, Zanini S (2014). Patterns of habituation and clinical fluctuations in migraine. Cephalalgia.

[13] Valeriani M, de Tommaso M, Restuccia D, Le Pera D, Guido M, Iannetti GD (2003). Reduced habituation to experimental pain in migraine patients: a CO(2) laser evoked potential study. Pain.

[14] Chen ACN, Niddam DM, Arendt-Nielsen L (2001). Contact heat evoked potentials as a valid means to study nociceptive pathways in human subjects. Neurosci Lett.

[15] Valeriani M, Le Pera D, Niddam D, Chen ACN, Arendt-Nielsen L (2002). Dipolar modelling of the scalp evoked potentials to painful contact heat stimulation of the human skin. Neurosci Lett.

[16] Lev R, Granovsky Y, Yarnitsky D (2010). Orbitofrontal disinhibition of pain in migraine with aura: An interictal EEG-mapping study. Cephalalgia.

[17] Lev R, Granovsky Y, Yarnitsky D (2013). Enhanced Pain Expectation in Migraine: EEG-Based Evidence for Impaired Prefrontal Function. Headache J Head Face Pain.

[18] Rolke R, Baron R, Maier C, Tolle TR, Treede RD, Beyer A (2006). Quantitative sensory testing in the German Research Network on Neuropathic Pain (DFNS): standardized protocol and reference values. Pain.

[19] Sand T, Nilsen KB, Hagen K, Stovner LJ (2010). Repeatability of cold pain and heat pain thresholds: The application of sensory testing in migraine research. Cephalalgia.

[20] Burstein R, Yarnitsky D, Goor-Aryeh I, Ransil BJ, Bajwa ZH (2000). An association between migraine and cutaneous allodynia. Ann Neurol.

[21] Teepker M, Peters M, Kundermann B, Vedder H, Schepelmann K, Lautenbacher S (2011). The Effects of Oral Contraceptives on Detection and Pain Thresholds As Well As Headache Intensity During Menstrual Cycle in Migraine. Headache J Head Face Pain.

[22] Suttrup I, Oberdiek D, Suttrup J, Osada N, Evers S, Marziniak M (2011). Loss of sensory function in patients with idiopathic hand dystonia. Mov Disord.

[23] Headache Classification Subcommittee of the International Headache Society (2004). The International Classification of Headache Disorders.

[24] de Tommaso M, Valeriani M, Sardaro M, Serpino C, Fruscolo OD, Vecchio E (2009). Pain perception and laser evoked potentials during menstrual cycle in migraine. J Headache Pain.

[25] Laux L, Glanzmann P, Schaffner P, Spielberger CD (1981). Das State-Trait-Angstinventar (STAI).

[26] de Tommaso M, Baumgartner U, Sardaro M, Difruscolo O, Serpino C, Treede R (2008). Effects of Distraction Versus Spatial Discrimination on Laser-Evoked Potentials in Migraine. Headache J Head Face Pain.

[27] Truini A, Galeotti F, Pennisi E, Casa F, Biasiotta A, Cruccu G (2007). Trigeminal small-fibre function assessed with contact heat evoked potentials in humans. Pain.

[28] Sachs L (2006). Utilized Statistics.

[29] Miller R (1981). Simultaneous Statistical Inference.

[30] Dupont WD, Plummer WD (1990). Power and Sample Size Calculations: A Review and Computer Program. Control Clin Trials.

[31] *Java-unterstützte Münsteraner Biometrie-Oberfläche*. [ http://campus.uni-muenster.de/fileadmin/einrichtung/imib/lehre/skripte/biomathe/bio/bio.html]

[32] Weissman-Fogel I, Sprecher E, Granovsky Y, Yarnitsky D (2003). Repeated noxious stimulation of the skin enhances cutaneous pain perception of migraine patients in-between attacks: clinical evidence for continuous sub-threshold increase in membrane excitability of central trigeminovascular neurons. Pain.

[33] Sand T, Zhitniy N, Nilsen KB, Helde G, Hagen K, Stovner LJ (2008). Thermal pain thresholds are decreased in the migraine preattack phase. Eur J Neurol.

[34] Ashkenazi A, Sholtzow M, Shaw J, Burstein R, Young W (2007). Identifying cutaneous allodynia in chronic migraine using a practical clinical method. Cephalalgia.

[35] Schwedt TJ, Krauss MJ, Frey K, Gereau RW (2011). Episodic and chronic migraineurs are hypersensitive to thermal stimuli between migraine attacks. Cephalalgia.

[36] Schoenen J (1996). Deficient habituation of evoked cortical potentials in migraine: a link between brain biology, behavior and trigeminovascular activation?. Biomed Pharmacother.

[37] Hansen JM, Bolla M, Magis D, de Pasqua V, Ashina M, Thomsen LL (2011). Habituation of evoked responses is greater in patients with familial hemiplegic migraine than in controls: a contrast with the common forms of migraine. Eur J Neurol.

[38] de Tommaso M, Lo Sito L, Di Fruscolo O, Sardaro M, Pia Prudenzano M, Lamberti P (2005). Lack of habituation of nociceptive evoked responses and pain sensitivity during migraine attack. Clin Neurophysiol.

[39] Gijsbers K, Nicholson F (2005). Experimental pain thresholds influenced by sex of experimenter. Percept Mot Skills.

